# A comparative machine and deep learning approach for predicting ultimate bearing capacity of shallow foundations in cohesionless soil

**DOI:** 10.1038/s41598-025-22812-7

**Published:** 2025-11-18

**Authors:** Mahmoud El Gendy

**Affiliations:** Department of Civil Engineering, Faculty of Engineering, Port Said University, Port Said, Egypt

**Keywords:** Artificial intelligence, Machine learning, Deep learning, Bearing capacity, Shallow foundations, Cohesionless soil, Civil engineering, Applied mathematics, Computational science

## Abstract

This study developed a Python-based framework to predict the ultimate bearing capacity of shallow foundations on cohesionless soil, employing machine learning (*ML*) and deep learning (*DL*) techniques. Utilizing a comprehensive dataset of 116 footing experiments, Eleven *ML* models (Gaussian Process Regression (*GPR*), Extreme Gradient Boosting (*XGBoost*), Gradient Boosting Machine (*GBM*), Random Forest (*RF*), Categorical Boosting (*CatBoost*) etc.) and five *DL* models (Artificial Neural Network (*ANN*), Deep Neural Network (*DNN*), etc.) trained and compared against traditional methods. Input parameters included foundation dimensions and soil properties. Results demonstrated that *ML* and *DL* models significantly outperformed traditional equations, achieving higher accuracy. Ensemble methods like *GPR*, *XGBoost*, *GBM*, *RF*, and *CatBoost* exhibited superior performance, with a Coefficient of Determination (*R*^2^) values above 0.988 and a Mean Absolute Percentage Error (*MAPE*) below 5.07%. Conversely, traditional methods showed lower accuracy, with *R*^2^ values ranging from 0.684 to 0.82 and *MAPE* exceeding 19.63%. Taylor diagram analysis confirmed the improved performance of *ML* and *DL*. Additionally, a SHapley Additive exPlanations (*SHAP*) analysis highlighted foundation depth and soil friction angle as the most influential parameters, consistent with geotechnical principles.

## Introduction

The ultimate bearing capacity (*q*_*u*_) and allowable settlement are crucial factors in designing shallow foundations. The ultimate bearing capacity is influenced by the soil’s shear strength and is typically estimated to be using theories developed by *Terzaghi*^1^, *Meyerhof*^2^, *Hansen*^3^, *Vesić*^4^, and others^5,6^. However, there are notable differences among the equations used to calculate bearing capacity. Additionally, these theories rely on several assumptions that simplify the complexities of the problem^7^.

Beyond analytical techniques for estimating bearing capacity, several scholars have investigated semi-empirical approaches for assessing foundation bearing capacity ). Although footing load tests provide the most accurate means of directly measuring the ultimate bearing capacity of foundations at a specific site, practical constraints related to cost and time often prevent their use. Typically, experimental studies are carried out on smaller-scale models in laboratory settings, which are considerably smaller than actual foundations^8^. Furthermore, Finite element method is a highly effective numerical technique for studying soil-structure interaction behavior. Numerous studies have utilized this technique to evaluate the ultimate bearing capacity of shallow foundations, and the accuracy of the results has been validated by comparisons with experimental data^9–11^.

The twenty-first century has witnessed the rapid expansion of Artificial Intelligence (AI), driven by its capacity to process distorted, incomplete, or fuzzy data. This ability to manage input uncertainty renders it a powerful tool for geotechnical problem-solving. Each AI method presents unique characteristics, benefits, and drawbacks, enabling researchers to explore diverse approaches to determine optimal solutions. AI techniques have been applied to a broad spectrum of geotechnical engineering areas, including deep foundations^12–16^, shallow foundations^17–22^, rock strength prediction^23,24^, slope stability^25,26^, soil strength forecasting^27–29^, landslide identification^30,31^, tunneling^32,33^, and soil liquefaction^34,35^. Furthermore, numerous studies have provided state-of-the-art reviews on AI applications in geotechnical engineering^36–41^.

Intelligent systems such as machine learning (*ML*), and deep learning (*DL*) are commonly employed to model complex interactions between inputs and outputs or to identify patterns within available data. AI-based methods excel in capturing inherent nonlinearity and intricate interactions among variables across various domains. These approaches can learn the relationships between soil mechanical properties, foundation geometry, and bearing capacity without needing prior knowledge of^42–44^. Recently, researchers have utilized various AI techniques to tackle the ultimate bearing capacity (*q*_*u*_) problem in shallow foundations. Techniques such as artificial neural networks (*ANN*)^45–51^, adaptive neuro-fuzzy inference systems (*ANFIS*)^50,52–54^, Deep Neural Network (*DNN*)^45,55–58^, Convolutional Neural Network (*CNN*)^59,60^, Recurrent Neural Networks (*RNN*)^59,61^, Feedforward Neural Networks (*FFNN*)^62,63^, Long short-term memory (*LSTM*)^59,60,64^, support vector regression (*SVR*)^20,56,58,65^, least squares *SVR* (*LSSVR*)^47,66,67^, random forests (*RF*)^59,68,69^, gradient boosting machine (*GBM*)^42,47^, gaussian process (*GPR*)^55,56,70,71^, k-nearest neighbors (*KNN*)^65^, decision tree (*DT*)^65,69,72,73^, ensemble tree (*ET*)^69,71–73^, Light Gradient Boosting Machine (*LightGBM*)^60,74,75^, Bagging Regressor (*BR*)^47,76^, Categorical Boosting (*CatBoost*)^77,78^, Ada Boost (*AdaBoost*)^72,79^, and extreme gradient boosting (*XGBoost*)^60,65,72^ have all shown success in estimating the ultimate bearing capacity of shallow foundations on soil.

This study develops a user-friendly Python framework to estimate the ultimate bearing capacity of shallow foundations, automating key steps and making advanced machine learning (*ML*) and deep learning (*DL*) techniques accessible to geotechnical professionals. Using a dataset of 116 footing experiments and input parameters like foundation dimensions and soil properties, the study trains and compare eleven *ML* models (including *GPR*, *XGBoost*, etc.) and five *DL* models (*ANN*, *CNN*, etc.) against traditional theoretical equations, aiming to improve the accuracy and efficiency of ultimate bearing capacity prediction.

## Methodology

This study employed a comprehensive approach, utilizing a robust and diverse dataset of 116 footing experiments, as listed inTable 1, compiled from a wide range of prior research. The primary goal was to develop advanced machine learning (*ML*) and deep learning (*DL*) models capable of accurately predicting the bearing capacity (*q*_*u*_) of shallow foundations in cohesionless soil. To achieve this, the study incorporated key factors identified through an extensive review of the literature. These factors, namely foundation width (*B*), foundation depth (*D*), length-to-width ratio (*L*/*B*), soil unit weight (γ), and internal friction angle (φ), served as the input variables. The output variable was the ultimate bearing capacity (*q*_*u*_). Understanding the influence of these parameters is crucial for accurate *q*_*u*_ prediction.

**Table 1:** Comparison of various *ML* algorithms.

*B* [m]	*D* [m]	*L*/*B* [-]	γ [kN/m^3^]	φ [°]	*q*_*u*_ [kN/m^2^]	RefrenceS
0.08	0	1	17.2	42.8	133	*Golder *et al*.*^90^
0.15	0	1	17.2	42.8	246	
0.05	0	1	17.2	42.8	109	*Eastwood* ^91^
0.08	0	1	17.1	42.8	130	
0.1	0	1	17.1	42.8	152	
0.15	0	1	17.1	42.8	214	
0.2	0	1	17.1	42.8	266	
0.25	0	1	17.1	42.8	333	
0.3	0	1	17.1	42.8	404	
0.03	0	1	15.89	42	52	*Subrahmanyam* ^92^
0.04	0	1	15.89	42	92	
0.05	0	1	15.89	42	95	
0.6	0.3	2	9.85	34.9	270	*Muhs *et al*.*^93^
0.6	0	2	10.2	37.7	200	
0.6	0.3	2	10.2	37.7	570	
0.6	0	2	10.85	44.8	860	
0.6	0.3	2	10.85	44.8	1760	
0.5	0	1	10.2	37.7	154	*Weiß* ^94^
0.5	0	1	10.2	37.7	165	
0.5	0	2	10.2	37.7	203	
0.5	0	2	10.2	37.7	195	
0.5	0	3	10.2	37.7	214	
0.52	0	3.85	10.2	37.7	186	
0.5	0.3	1	10.2	37.7	681	
0.5	0.3	2	10.2	37.7	542	
0.5	0.3	2	10.2	37.7	530	
0.5	0.3	3	10.2	37.7	402	
0.52	0.3	3.85	10.2	37.7	413	
0.5	0	1	11.7	37	111	*Muhs and Weiß* ^95^
0.5	0	1	11.7	37	132	
0.5	0	2	11.7	37	143	
0.5	0.013	1	11.7	37	137	
0.5	0.029	4	11.7	37	109	
0.5	0.127	4	11.7	37	187	
0.5	0.3	1	11.7	37	406	
0.5	0.3	1	11.7	37	446	
0.5	0.3	4	11.7	37	322	
0.5	0.5	2	11.7	37	565	
0.5	0.5	4	11.7	37	425	
0.5	0	1	12.41	44	782	
0.5	0	4	12.41	44	797	
0.5	0.3	1	12.41	44	1940	
0.5	0.3	1	12.41	44	2266	
0.5	0.5	2	12.41	44	2847	
0.5	0.5	4	12.41	44	2033	
0.5	0.49	4	12.27	42	1492	
0.5	0	1	11.77	37	123	
0.5	0	2	11.77	37	134	
0.5	0.3	1	11.77	37	370	
0.5	0.5	2	11.77	37	464	
0.5	0	4	12	40	461	
0.5	0.5	4	12	40	1140	
1	0.2	3	11.97	39	710	*Muhs and Weiß* ^96^
1	0	3	11.93	40	630	
1	0.71	1	15.5	35.3	1550	*Briaud and Gibbens* ^97^
1.5	0.76	1	15.5	35.3	1355.56	
2.5	0.76	1	15.5	35.3	1152	
3	0.76	1	15.5	35.3	1144.44	
3	0.89	1	15.5	35.3	1011.11	
0.991	0.711	1	15.8	32	1773.7	*Briaud and Gibbens* ^98^
3.004	0.762	1	15.8	32	1019.4	
2.489	0.762	1	15.8	32	1158	
1.492	0.762	1	15.8	32	1540	
3.016	0.889	1	15.8	32	1161.2	
0.0585	0.029	5.95	15.7	34	58.5	*Gandhi* ^99^
0.0585	0.058	5.95	15.7	34	70.91	
0.0585	0.029	5.95	16.1	37	82.5	
0.0585	0.058	5.95	16.1	37	98.93	
0.0585	0.029	5.95	16.5	39.5	121.5	
0.0585	0.058	5.95	16.5	39.5	142.9	
0.0585	0.029	5.95	16.8	41.5	157.5	
0.0585	0.058	5.95	16.8	41.5	184.9	
0.0585	0.029	5.95	17.1	42.5	180.5	
0.0585	0.058	5.95	17.1	42.5	211	
0.094	0.047	6	15.7	34	74.7	
0.094	0.094	6	15.7	34	91.5	
0.094	0.047	6	16.1	37	104.8	
0.094	0.094	6	16.1	37	127.5	
0.094	0.047	6	16.5	39.5	155.8	
0.094	0.094	6	16.5	39.5	185.6	
0.094	0.047	6	16.8	41.5	206.8	
0.094	0.094	6	16.8	41.5	244.6	
0.094	0.047	6	17.1	42.5	235.6	
0.094	0.094	6	17.1	42.5	279.6	
0.152	0.075	5.95	15.7	34	98.2	
0.152	0.15	5.95	15.7	34	122.3	
0.152	0.075	5.95	16.1	37	143.3	
0.152	0.15	5.95	16.1	37	176.4	
0.152	0.075	5.95	16.5	39.5	211.2	
0.152	0.15	5.95	16.5	39.5	254.5	
0.152	0.075	5.95	16.8	41.5	285.3	
0.152	0.15	5.95	16.8	41.5	342.5	
0.152	0.075	5.95	17.1	42.5	335.3	
0.152	0.15	5.95	17.1	42.5	400.6	
0.094	0.047	1	15.7	34	67.7	
0.094	0.094	1	15.7	34	90.5	
0.094	0.047	1	16.1	37	98.8	
0.094	0.094	1	16.1	37	131.5	
0.094	0.047	1	16.5	39.5	147.8	
0.094	0.094	1	16.5	39.5	191.6	
0.094	0.047	1	16.8	41.5	196.8	
0.094	0.094	1	16.8	41.5	253.6	
0.094	0.047	1	17.1	42.5	228.8	
0.094	0.094	1	17.1	42.5	295.6	
0.152	0.075	1	15.7	34	91.2	
0.152	0.15	1	15.7	34	124.4	
0.152	0.075	1	16.1	37	135.2	
0.152	0.15	1	16.1	37	182.4	
0.152	0.075	1	16.5	39.5	201.2	
0.152	0.15	1	16.5	39.5	264.5	
0.152	0.075	1	16.8	41.5	276.3	
0.152	0.15	1	16.8	41.5	361.5	
0.152	0.075	1	17.1	42.5	325.3	
0.152	0.15	1	17.1	42.5	423.6	
0.06	0	1	14.8	42	72	*Cerato and Lutenegger* ^87^
0.06	0	1	15.4	42	106	

This study utilized eleven machine learning (*ML*) models: Gaussian Process (*GPR*), Extreme Gradient Boosting (*XGBoost*), Light Gradient Boosting Machine (*LGBM*), Gradient Boosting Machine (*GBM*), Random Forest (*RF*), Categorical Boosting (*CatBoost*), Ada Boost (*AdaBoost*), K-Nearest Neighbors (*KNN*), Bagging Regressor (*BR*), Decision Tree (*DT*), and Support Vector Regression (*SVR*). Additionally, five deep learning (*DL*) models were used: Artificial Neural Network (*ANN*), Deep Neural Network (*DNN*), Convolutional Neural Network (*CNN*), Recurrent Neural Networks (*RNN*), and Feedforward Neural Network (*FFNN*). All models were implemented using the Python programming environment, alongside well-known equations by *Terzaghi*, *Meyerhof*, *Vesić*, *Hansen*, Eurocode 7 (*EC*7), and Egyptian Code (*ECP*).

### Soft computing approaches

Machine learning (*ML*) and deep learning (*DL*) models are a core component of artificial intelligence, focusing on algorithms that learn from data to make predictions without explicit programming. I utilized a range of these models, broadly categorized into ensemble methods and traditional *ML* algorithms. The ensemble methods, including Gaussian Process Regressor (*GPR*), Extreme Gradient Boosting (*XGBoost*), Light Gradient Boosting Machine (*LightGBM*), Gradient Boosting Machines (*GBM*), Random Forest (*RF*), Categorical Boosting (*CatBoost*), Adaptive Boosting (*AdaBoost*), K-Nearest Neighbors (*KNN*), Bagging Regressor (*BR*), Decision Trees (*DT*), Support Vector Machine (*SVM*), Artificial Neural Network (*ANN*), Deep Neural Network (*DNN*), Convolutional Neural Network (*CNN*), Recurrent Neural Network (*RNN*), and Feedforward Neural Network (*FFNN*) to comprehensively evaluate various approaches to predicting ultimate bearing capacity.

A comparison of various *ML* and *DL* algorithms, including their type, strengths, weaknesses, best use cases, performance and computational cost are listed in Table 1 and Table 2, respectively.

**Table 2:** Comparison of various *DL* algorithms.

Algorithm	Architecture	Strengths	Weaknesses	Use sases	Performance
*ANN* ^82–84^	Input layer, one or more hidden layers, output layer. Fully connected	Versatile and flexible. Can model complex, non-linear relationships	Computationally expensive. Prone to overfitting. Black-box nature	General-purpose tasks like classification, regression, and clustering	High accuracy for small to medium datasets, but slow for large datasets
*DNN* ^85,60^	Multiple hidden layers between input and output. Fully connected	Hierarchical feature learning. State-of-the-art performance in many domains	Computationally expensive. Requires large datasets. Hard to interpret	Image recognition, NLP, speech recognition, and complex pattern recognition	State-of-the-art performance for large datasets, but resource intensive
*CNN* ^59,60^	Convolutional layers, pooling layers, fully connected layers. Local connectivity	Excellent for spatial data (e.g., images). Reduces parameters via weight sharing	Computationally expensive. Requires large datasets. Limited to grid-like data	Image classification, object detection, video analysis, and medical imaging	State-of-the-art performance for image and video data
*RNN* ^59,61^	Recurrent connections with loops. Hidden state to capture temporal dependencies	Handles sequential data. Models temporal dependencies effectively	Suffers from vanishing/exploding gradients. Computationally expensive. Computationally expensive	Time-series forecasting, NLP, speech recognition, and video analysis	High accuracy for sequential data, but slower than CNNs and FFNNs
*FFNN* ^62,63^	Input layer, hidden layers, output layer. No cycles or loops	Simple and easy to implement. Handles static data well	Cannot model sequential data. Prone to overfitting. Limited to small datasets	Classification, regression, and pattern recognition for static data	Good for small datasets but struggle with large or sequential data

### Theoretical bearing capacity equations

*Terzaghi*^1^, applied *Prandtl’s*^86^ plastic failure theory to formulate a method for determining the bearing capacity of shallow foundations, considering soil cohesion, effective stress, and the angle of internal friction. Later, *Meyerhof*^2^, *Hansen*^3^, *Vesić*^4^ The European Code (*EC*7: Geotechnical Design^6^, and the Egyptian Code for Soil Mechanics and Foundation Design and Implementation (*ECP*-202: Part 4-Deep Foundations^5^ extended Terzaghi’s equation by incorporating shape and depth factors, among others. As shown in Table 3, the fundamental structure of these classical equations remained consistent with Terzaghi’s. However, these traditional methods, despite being supported by extensive in situ and laboratory data, are limited by their reliance on simplifying assumptions, leading to potential inaccuracies in bearing capacity predictions^87,88^.

1 $$q_{u} = cN_{c} s_{c} d_{c} + \gamma DN_{q} s_{q} d_{q} + \frac{1}{2}\gamma BN_{\gamma } s_{\gamma } d_{\gamma }$$

2 $$K_{p} = \frac{1 + \sin \varphi }{{1 - \sin \varphi }}$$

**Table 3:** Well known theoretical equations for the bearing capacity of shallow foundations.

Reference	Bearing capacity factor	Shape and depth factor
*Terzaghi* ^1^	$a = e^{{\left( {0.75\pi - \frac{\varphi }{2}} \right)\tan \varphi }}$ $N_{q} = \frac{{a^{2} }}{{a\cos^{2} \left( {45 + \frac{\varphi }{2}} \right)}}$ $N_{c} = \left( {N_{q} - 1} \right)\cot \varphi$ $N_{\gamma } = \frac{\tan \varphi }{2}\left( {\frac{{K_{p} }}{{\cos^{2} \varphi }} - 1} \right)$	No shape or depth factor
*Meyerhof* ^2^	$N_{q} = e^{\pi \tan \varphi } \tan^{2} \left( {45 + \frac{\varphi }{2}} \right)$ $N_{c} = \left( {N_{q} - 1} \right)\cot \varphi$ $N_{\gamma } = \left( {N_{q} - 1} \right)\tan \left( {1.4\varphi } \right)$	$s_{c} = 1 - 0.2K_{p} \frac{B}{L}$ $$\left\{ {\begin{array}{*{20}c} {{\text{For}} \varphi = 0 \to s_{q} = s_{\gamma } = 1} \\ {{\text{For}} \varphi \ge 10 \to s_{q} = s_{\gamma } = 1 + 0.1K_{p} \frac{B}{L}} \\ \end{array} } \right.$$ $d_{c} = 1 + 0.2\sqrt {K_{p} } \frac{D}{B}$ $$\left\{ {\begin{array}{*{20}c} {{\text{For}} \varphi = 0 \to d_{q} = d_{\gamma } = 1} \\ {{\text{For}} \varphi \ge 10 \to d_{q} = d_{\gamma } = 1 + 0.1\sqrt {K_{p} } \frac{D}{B}} \\ \end{array} } \right.$$
*Hansen* ^3^	$N_{q} = e^{\pi \tan \varphi } \tan^{2} \left( {45 + \frac{\varphi }{2}} \right)$ $N_{c} = \left( {N_{q} - 1} \right)\cot \varphi$ $N_{\gamma } = 1.5\left( {N_{q} - 1} \right)\tan \varphi$	$s_{c} = 1 + \left( {\frac{{N_{q} }}{{N_{c} }}} \right)\left( \frac{B}{L} \right)$ $1 + \left( \frac{B}{L} \right)\tan \varphi$ $s_{\gamma } = 1 - 0.4\left( \frac{B}{L} \right)$ $d_{\gamma } = 1$ $$\left\{ {\begin{array}{*{20}c} {{\text{For}} D \le B \to \left\{ {\begin{array}{*{20}c} {d_{q} = 1 + 2\tan \varphi \left( {1 - \sin \varphi } \right)^{2} \left( \frac{D}{B} \right)} \\ {d_{c} = 1 + 0.4\frac{D}{B}} \\ \end{array} } \right.} \\ {{\text{For}} D > B \to \left\{ {\begin{array}{*{20}c} {d_{q} = 1 + 2\tan \varphi \left( {1 - \sin \varphi } \right)^{2} \tan^{ - 1} \left( \frac{D}{B} \right)} \\ {d_{c} = 1 + 0.4\tan^{ - 1} \left( \frac{D}{B} \right)} \\ \end{array} } \right.} \\ \end{array} } \right.$$
*Vesić* ^4^	$N_{q} = e^{\pi \tan \varphi } \tan^{2} \left( {45 + \frac{\varphi }{2}} \right)$ $N_{c} = \left( {N_{q} - 1} \right)\cot \varphi$ $N_{\gamma } = 2\left( {N_{q} + 1} \right)\tan \varphi$	
*EC*7^6^	$N_{q} = e^{\pi \tan \varphi } \tan^{2} \left( {45 + \frac{\varphi }{2}} \right)$ $N_{c} = \left( {N_{q} - 1} \right)\cot \varphi$ $N_{\gamma } = 2\left( {N_{q} - 1} \right)\tan \varphi$	$s_{q} = 1 + \left( \frac{B}{L} \right)\sin \varphi$ $s_{\gamma } = 1 - 0.3\left( \frac{B}{L} \right)$ $s_{c} = \left( {\frac{{s_{q} N_{q} - 1}}{{N_{q} - 1}}} \right)$ No depth factor
*ECP* ^5^		$s_{c} = s_{q} = 1 + 0.3\left( \frac{B}{L} \right)$ $s_{\gamma } = 1 - 0.3\left( \frac{B}{L} \right)$ No depth factor

Were ${q}_{u}$ ultimate bearing capacity of footing; *c* soil cohesion; $\gamma$ effective unit weight of the soil below and around the foundation; *B* footing width; *L* footing length; *D* foundation depth; ${N}_{c}$, ${N}_{q}$, and ${N}_{\gamma }$ non-dimensional bearing capacity factors as exponential functions of $\varphi$; $\varphi$ internal friction angle; ${s}_{c}$, ${s}_{q}$, and ${s}_{\gamma }$ non-dimensional shape factors; ${d}_{c}$, ${d}_{q}$, and ${d}_{\gamma }$ non-dimensional depth factors; and ${K}_{p}$ is the passive earth pressure.

## Database and modeling

### Input selection

For shallow foundations on granular soils, numerous studies emphasize the importance of foundation geometry (width *B*, length *L*, depth *D*), soil friction angle (φ), and unit weight (γ) in determining ultimate bearing capacity. These factors directly influence stress distribution and failure mechanisms, which are crucial for solving bearing capacity problems. Foundation depth (*D*) significantly impacts bearing capacity, while soil friction angle (φ) is considered the most influential parameter. Uncertainty analyses by *Foye *et al*.*^89^ reinforce the key roles of *B*, *L*, *D*, φ, and γ. Consequently, this study employs these parameters to predict the ultimate bearing capacity of shallow foundations on granular soil.

### Dataset

**Fig. 1:**
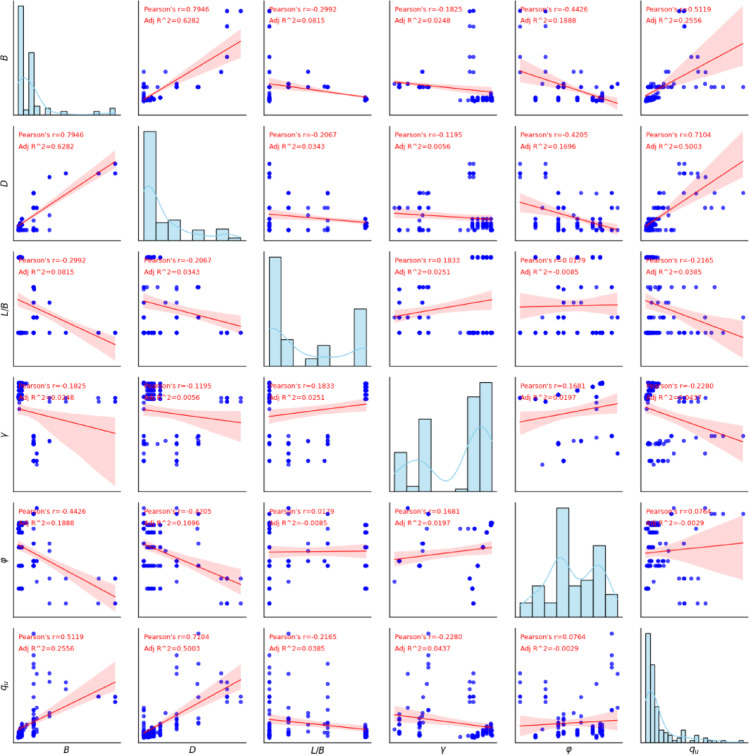
Correlation matrix for the bearing capacity databases.

Data quantity and quality are essential for improving modeling accuracy. This study utilized 116 data points sourced from publications by *Golder *et al*.*^90^, *Eastwood*^91^, *Subrahmanyam*^92^, *Muhs *et al*.*^93^, *Weiß*^94^, *Muhs and Weiß*^95,96^, *Briaud and Gibbens*^97,98^, *Gandhi*^99^, and *Cerato and Lutenegger*^87^. The dataset comprised 64 small-scale and 52 large-scale experiments. Table 4 comprehensively lists the literature sources and parameter ranges for each footing. Despite variations in testing conditions, the combined dataset provides a substantial volume of diverse data, reflecting various simulations of footing bearing capacity in cohesionless soil in real-world scenarios. Statistical characteristics and the distribution charts of the values of different parameters in the database are depicted in Fig. 1 and summarized in Table 5.

**Table 4:** Database for prediction of the ultimate bearing capacity.

Algorithm	Type	Strengths	Weaknesses	Best use cases	Performance	Computational cost
*GPR* ^70,56^	Probabilistic	Probabilistic predictions with uncertainty estimates. Handles non-linear relationships. Strong theoretical foundation	Computationally expensive. Sensitive to kernel choice. Poor scalability for large datasets	Small datasets with uncertainty quantification. Scientific modeling, Bayesian optimization	High accuracy for small datasets, but slow for large datasets	High
*XGBoost* ^80,60^	Ensemble (Boosting)	High accuracy and scalability. Handles missing values. Regularization to prevent overfitting	Requires careful hyperparameter tuning. Computationally expensive for very large datasets	Large datasets, complex prediction tasks	State-of-the-art performance for structured data	High
*LGBM* ^74,75^	Ensemble (Boosting)	Extremely fast and memory efficient. Handles large datasets. Supports categorical features	May overfit on small datasets. Requires tuning for optimal performance	Large datasets, high-dimensional data, ranking tasks	Faster than XGBoost, with comparable accuracy	Moderate
*GBM* ^42,47^	Ensemble (Boosting)	High accuracy. Handles non-linear relationships. Robust to outliers	Computationally expensive. Prone to overfitting without tuning	Diverse datasets, complex prediction tasks	High accuracy but slower than XGBoost and LGBM	High
*RF* ^42,69^	Ensemble (Bagging)	Robust to overfitting. Handles high-dimensional data. Provides feature importance	Less interpretable than single trees. Computationally expensive for large datasets	Diverse datasets, feature selection	High accuracy, but slower than boosting methods	Moderate to High
*CatBoost* ^77,78^	Ensemble (Boosting)	Handles categorical data natively. Robust to overfitting. GPU support for fast training	Requires tuning. Less flexible for custom loss functions	Tabular data with categorical features, click-through rate prediction	High accuracy, especially for categorical data	High
*AdaBoost* ^79,72^	Ensemble (Boosting)	Improves weak learners. Simple to implement. Robust to overfitting	Sensitive to noisy data. Computationally expensive for large datasets	Binary classification, face detection	Good accuracy for small datasets but struggles with noise	Moderate
*KNN* ^65^	Instance-based	Simple and intuitive. No training phase. Handles non-linear data	Computationally expensive for large datasets. Sensitive to feature scaling. Struggles with high-dimensional data	Image recognition, recommendation systems	Good for small datasets, but slow for large datasets	Moderate to High
*BR* ^47,76^	Ensemble (Bagging)	Reduces variance and overfitting. Combines multiple models for robustness. variance, improves generalization, robust to noise	Computationally expensive. Limited bias reduction	Regression tasks, noise reduction	Improves stability and accuracy of base models	Moderate to High
*DT* ^73,65^	Non-parametric	Easy to interpret and visualize. Handles mixed data types. No need for feature scaling	Prone to overfitting. High variance. Struggles with extrapolation	Classification and regression, interpretable models	Good for small datasets, but prone to overfitting	Low to Moderate
*SVR* ^55,81^	Kernel-based	Handles non-linear relationships using kernels. Robust to outliers. Provides probabilistic predictions	Computationally expensive. Requires careful hyperparameter tuning. Poor scalability for large datasets	Regression tasks with non-linear relationships, outlier-resistant modeling	High accuracy for small datasets, but slow for large datasets	High

**Table 5:** Statistics of input parameters.

	Min	Max	Mean	Std	Skewness	Kurtosis
*B* [m]	0.03	3.016	0.445147	0.629536	2.96819	8.769519
*D* [m]	0	0.89	0.171724	0.230613	1.662777	1.779513
*L*/*B* [-]	1	6	2.756034	2.105047	0.653817	− 1.32051
γ [kN/m^3^]	9.85	17.2	14.52698	2.544163	− 0.59387	− 1.31833
φ [°]	32	44.8	38.89052	3.352584	− 0.16356	− 0.93236
*q*_*u*_ [kN/m^2^]	52	2847	441.8961	524.2588	2.252382	5.028811

To facilitate model training and unbiased evaluation, the dataset was partitioned into two distinct subsets: a training set, comprising 70% of the data, and a testing set, comprising the remaining 30%. The training set was utilized to construct the *ML* and *DL* models, whereas the testing set, held separate during model development, was exclusively used to assess the models’ predictive performance on unseen data.

### Correlation analysis

Figure 1 provides a detailed visual and statistical analysis of the relationships between variables. The diagonal plots, featuring histograms with fitted curves, show the individual distribution of each variable. The off-diagonal scatter plots illustrate the linear relationship between pairs of variables, with a red regression line to highlight the trend^72^. Crucially, each of these scatter plots includes two key metrics: Pearson’s r, which measures the strength and direction of the linear correlation (e.g., the strong positive relationship between *B* and *D* with an r of 0.9383), and *R*^2^, which indicates how well the regression line fits the data. Analyzing these values reveals important insights, such as the moderate negative correlation between *B* and φ (*r*≈ − 0.4426) and the very weak relationship between *L*/*B* and γ (*r*≈0.1813). The plots in the final row, which compare all other variables against *q*_*u*_, are particularly useful for understanding which factors are most influential. Overall, this single visualization effectively summarizes the data, allowing for a quick and clear assessment of variable dependencies.

**Fig. 2:**
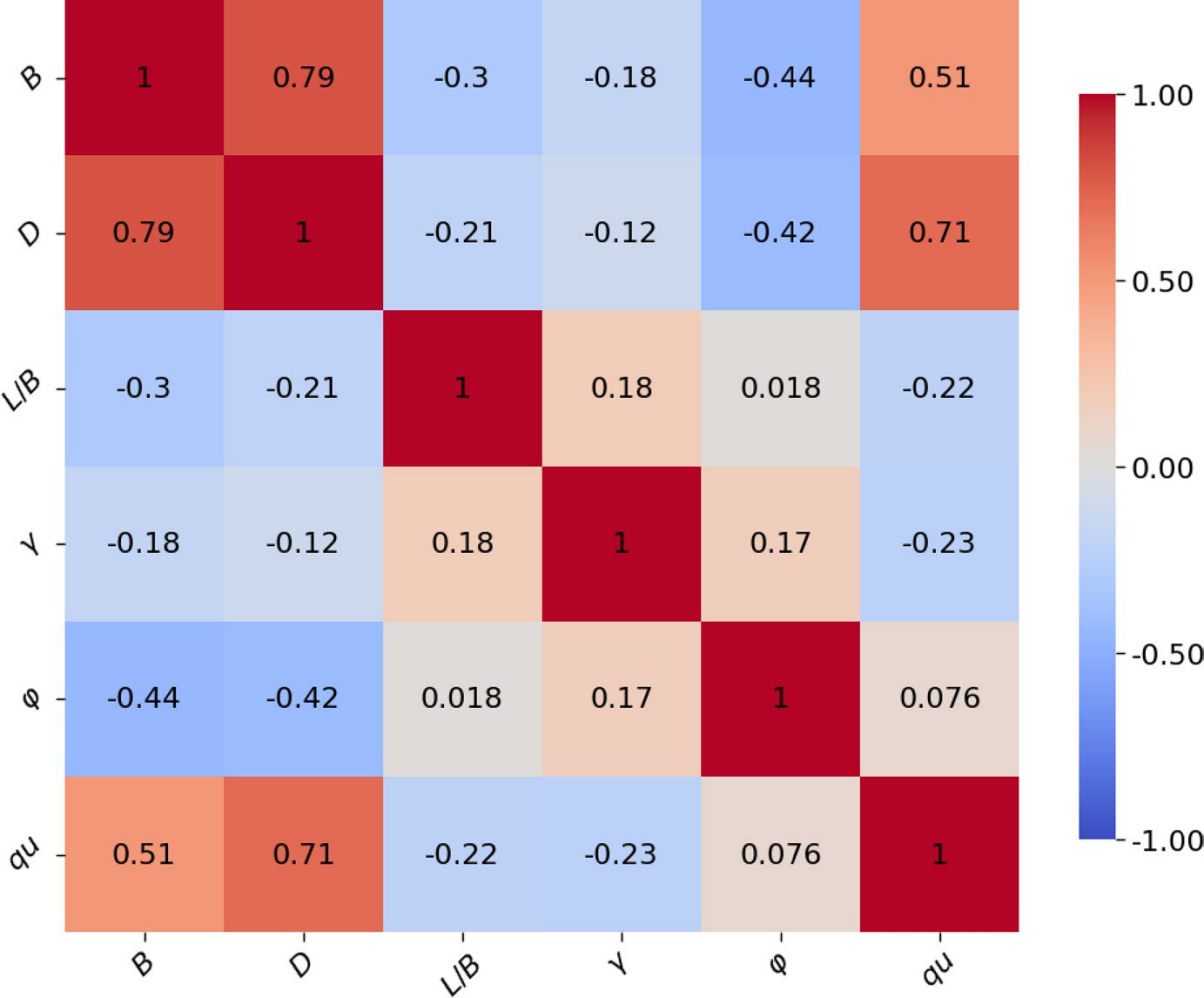
Heatmap correlation matrix.

Figure 2 illustrates the correlation matrix as a heatmap, providing a quantitative assessment of the relationships between *B*, *D*, *L*/*B*, γ, φ, and *q*_*u*_. The intensity of the heatmap’s colors corresponds to the magnitude of the correlation coefficients, with darker red indicating strong positive correlations and darker blue representing strong negative correlations. The diagonal elements naturally show a perfect correlation for each variable with itself.

The correlation between *B* (foundation width) and *D* (foundation depth) is the strongest (0.79), indicating that wider footings tend to have greater foundation depths. The correlation with *q*_*u*_ (ultimate bearing capacity) is also strong (0.51), suggesting that an increase in foundation width is strongly associated with an increase in the ultimate bearing capacity of the footing. The correlations with *L*/*B* (length *L* to width *B*) and φ (soil friction angle) are moderately negative at (− 0.3) and (− 0.44), respectively, while γ (unit weight) has the weakest correlation at (− 0.18).

*D* shows weak correlations with other variables, except with *B*, as already mentioned, and a strong positive correlation with *q*_*u*_ at (0.71), indicating that an increase in foundation depth is strongly associated with an increase in the ultimate bearing capacity of the footing. The correlations with *L*/*B* and γ are moderately negative at (− 0.21) and (− 0.12), respectively, while φ has a weaker correlation at (− 0.42).

*L*/*B* has a slight positive correlation with γ (0.18), and its correlation with φ is nearly zero (0.018), suggesting no significant linear relationship. Additionally, the correlation with *q*_*u*_ is weakly negative (− 0.22), indicating that longer footings tend to have slightly lower ultimate bearing capacity, in addition to the previously mentioned correlations with *B* and *D*.

γ shows a slight positive correlation with φ (0.17). Furthermore, the correlation with *q*_*u*_ is weakly negative (− 0.23), Besides the previously mentioned correlations with *B*,* D,* and *L*/*B*.

Besides the already mentioned correlations with *B*,* D*,* L*/*B*, and γ, the correlation between φ and *q*_*u*_ is nearly zero at (0.079), suggesting no significant linear relationship.

In summary, *q*_*u*_, the ultimate bearing capacity of the footing, exhibits various levels of correlation with the input variables. The strongest positive correlation is with *B* at (0. 79), highlighting the significant impact of the footing width on the ultimate bearing capacity. *q*_*u*_ also shows strong correlations with *D* (0.71), indicating that footing with greater depths tends to have higher ultimate bearing capacity. Correlations between *q*_*u*_ with both *L*/*B* and γ are weakly negative (− 0.22) and (− 0.23), respectively, while φ is nearly zero at (0.079), suggesting no significant linear relationship.

### Tuning hyperparameters of machine learning models

To enhance machine learning model performance, this study employed Bayesian optimization (*BO*) for hyperparameter tuning, addressing the limitations of traditional grid and random search methods. Specifically, a fivefold cross-validation (*CV*) integrated with *BO* (*BO* + 5*CV*) was implemented to mitigate overfitting and ensure robust, generalizable predictions. Utilizing Optuna’s Bayesian optimization, the process aimed to maximize the cross-validated *R*^2^ score by iteratively exploring hyperparameter combinations for parameters such as n_estimators, learning_rate, and depth across 1000 iterations. This approach defined an objective function that trains the model with suggested hyperparameters, evaluates its performance through fivefold *CV*, and returns the mean *R*^2^, ultimately identifying the optimal hyperparameter set and corresponding maximum *R*^2^ value, as detailed in Table 6.

**Table 6:** Optimal hyperparameters of machine learning models.

*ML*/*DL* Model	Optimal hyperparameters
XGBoost	n_estimators = 1172, learning_rate = 0.19767316614730276
LightGBM	n_estimators = 1482, learning_rate = 0.6992380456312691
GBM	n_estimators = 1087, learning_rate = 0.28980775639493667
RF	n_estimators = 108, max_depth = 15, min_samples_split = 2, min_samples_leaf = 1, bootstrap = False
CATBoost	iterations = 1000, learning_rate = 0.1, depth = 6, verbose = 0
AdaBoost	n_estimators = 241, learning_rate = 0.09257405215270152, loss = ‘square’, max_depth = 10, min_samples_split = 4, min_samples_leaf = 1
KNN	n_neighbors = 5, weights = ‘distance’, p = 1, algorithm = ‘auto’
BR	n_estimators = 97, max_samples = 0.9996785133314219, max_features = 0.8255801182352335, bootstrap = False, bootstrap_features = False, max_depth = 12, min_samples_split = 2, min_samples_leaf = 1
DT	max_depth = 8, min_samples_split = 2, min_samples_leaf = 1, max_features = None, splitter = best, criterion = absolute_error
SVM	kernel = rbf, C = 75.62164653446044, epsilon = 0.025523589047330056, degree = 3
ANN	hidden_layer_sizes = 10, activation = logistic, max_iter = 100,000, solver = lbfgs, random_state = 42
DNN	n_layers = 3, units_0 = 113, units_1 = 169, units_2 = 254, learning_rate = 0.002089738484827969, dropout_rate = 0.003010226821045029
CNN	n_conv_layers = 2, filters_0 = 106, filters_1 = 121, kernel_size = 4, learning_rate = 0.003535056792769612, dropout_rate = 0.041472436005474664
RNN	K-Fold MSE Scores: [np.float64(2.265767), np.float64(5.107743), np.float64(4.706106), np.float64(2.874644), np.float64(1.123151)] Mean K-Fold MSE: 3.215483 Best Validation MSE: 1.123151
FFNN	n_layers = 2, units_0 = 43, units_1 = 57, learning_rate = 0.008968117531970703, dropout_rate = 4.784803011470551e-05

The skopt.gp_minimize method was utilized for hyperparameter tuning of a Gaussian Process Regressor (*GPR*) to optimize its intricate kernel function. The objective was to minimize the negative mean squared error obtained from cross-validation. This approach, called Bayesian Optimization, effectively explores the optimal combinations of parameters within the kernel’s components.

The Recurrent Neural Network (*RNN*) model does not include hyperparameter tuning. It evaluates a single, fixed set of hyperparameters for an *LSTM* model using k-fold cross-validation. While it saves the best-performing model from those cross-validation runs, it does not systematically search for the optimal values for parameters like the number of *LSTM* units, epochs, or batch size. This makes it a model evaluation script rather than a hyperparameter optimization one.

### Model evaluation

Evaluating machine and deep learning models for predicting complex outcomes, like foundation bearing capacity, requires a comprehensive approach. It’s not enough to just train a model; you must also verify its ability to generalize new, unseen data using a testing dataset. This ensures the model’s predictions are reliable for real-world applications. A robust evaluation combines both visual diagnostics, such as scatter plots, to identify systematic errors, and quantitative metrics to precisely measure performance. The standard metrics used include the Coefficient of Determination (*R*^2^) to assess how well the model fits the data; Mean Absolute Percentage Error (*MAPE*), Mean Absolute Error (*MAE*), and Root Mean Square Error (*RMSE*) to quantify the magnitude of prediction errors; Mean Bias Error (*MBE*) to reveal any prediction bias. Additionally, metrics like the A20 Index provide the percentage of predictions within an acceptable ± 20% error margin, while the Scatter Index (*SI*) and Agreement Index (*d*) offer more normalized and robust measures of overall model accuracy and reliability. The A20 Index directly measures a model’s reliability by indicating the percentage of predictions that fall within a precise ± 20% error margin, with higher values showing a greater number of reliable predictions. The *SI* quantifies the dispersion of predictions relative to actual values; a low value signifies highly precise predictions with minimal scatter. Lastly, the Agreement Index (*d*) assesses the overall agreement between predicted and observed values on a scale from 0 to 1, where a value closer to 1, reflects a strong alignment between the model’s output and real-world data. Together, these methods provide a thorough and reliable assessment of a model’s predictive capabilities^78,89^. These metrics are defined as follows:

3 $$R^{2} = 1 - \frac{{\sum \left( {y_{i} - \hat{y}} \right)^{2} }}{{\sum \left( {y_{i} - \overline{y}} \right)^{2} }}$$

4 $$MAPE = \frac{100}{n}\mathop \sum \limits_{i = 1}^{n} \frac{{y_{i} - \hat{y}}}{{y_{i} }}$$

5 $$MAE = \frac{1}{n}\mathop \sum \limits_{i = 1}^{n} \left| {y_{i} - \hat{y}} \right|$$

6 $$MBE = \frac{1}{n}\mathop \sum \limits_{i = 1}^{n} \left( {y_{i} - \hat{y}} \right)$$

7 $$RMSE = \sqrt {\frac{1}{n}\sum \left( {y_{i} - \hat{y}} \right)^{2} }$$

8 $$CC = \frac{{\sum \left( {y_{i} - \overline{y}} \right)\left( {\hat{y} - \mathop y\limits^{ = } } \right)}}{{\sqrt {\sum \left( {y_{i} - \overline{y}} \right)^{2} \sum \left( {\hat{y} - \mathop y\limits^{ = } } \right)^{2} } }}$$

9 $${\text{A}}20 = \frac{1}{n}\mathop \sum \limits_{i = 1}^{n} \left\{ {\begin{array}{*{20}c} {1, {\text{if}} \left| {\frac{{\left( {y_{i} - \overline{y}} \right)}}{{y_{i} }}} \right| \le 0.2} \\ {0, otherwise } \\ \end{array} } \right.$$

10 $$SI = \frac{RMSE}{{\overline{y}}}$$

11 $$d = 1 - \frac{{\mathop \sum \nolimits_{i = 1}^{n} \left( {y_{i} - \hat{y}} \right)^{2} }}{{\mathop \sum \nolimits_{i = 1}^{n} \left( {\left| {\hat{y} - \overline{y}} \right| + \left| {y_{i} - \overline{y}} \right|} \right)^{2} }}$$

where ${y}_{i}$ is the actual value, $\widehat{y}$ is predicted value, $\overline{y }$ is the mean of the actual values, and $\overline{\overline{y}}$ is the mean of the predicted values.

Also, Taylor diagrams were used to provide a detailed statistical comparison between predicted and observed values, incorporating metrics such as correlation, *RMSE*, and normalized standard deviation. This allowed for a more nuanced understanding of model performance and error sources. To ensure robustness, uncertainty analysis was also incorporated.

A comprehensive evaluation framework, encompassing both visual and quantitative analyses, allows for a thorough and balanced assessment of model performance. This robust approach validates the scientific integrity of predictions and ensures their practical applicability in real-world scenarios.

## Results

### Performance and results of ML and DL models

**Fig. 3:**
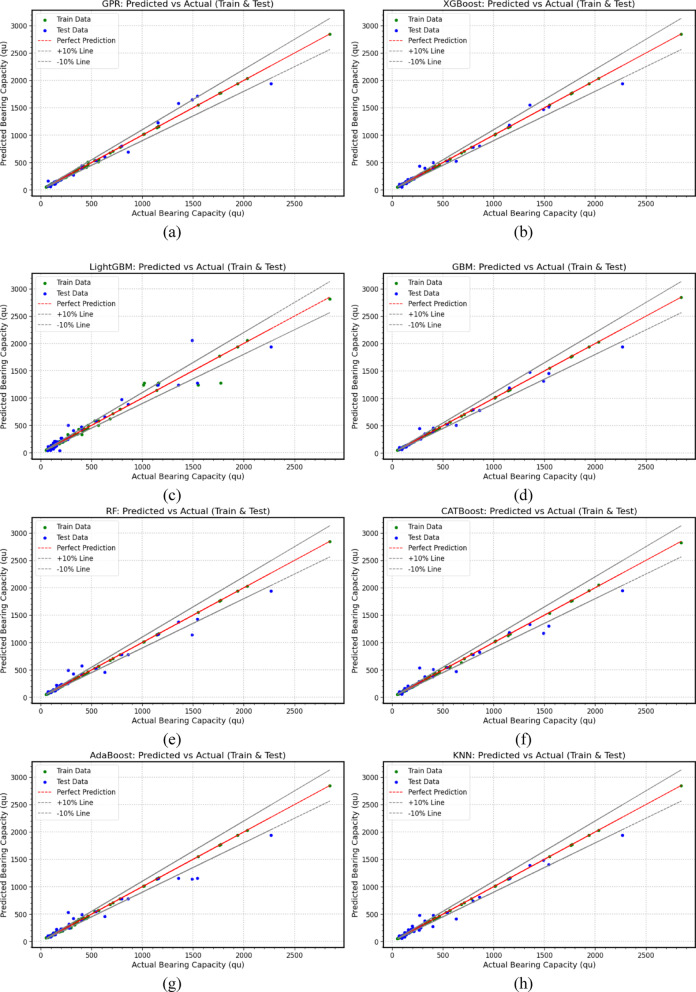
Scatter plots between actual and predicted ultimate bearing capacity values based on (**a**) *GPR*, (**b**) *XGBoost*, (**c**) *LightGBM*, (**d**) *GBM*, (**e**) *RF*, (**f**) *CATBoost*, (**g**) *AdaBoost*, (**h**) *KNN.*

The performance of various *ML* and *DL* models in predicting ultimate bearing capacity for footings with different cross-sectional shapes in sand is visualized in scatter plots, Fig. 3 through Fig. 5. The close clustering of training (70%) and testing (30%) data points around the diagonal line for the *ML* and *DL* models indicates strong agreement between predictions and experimental results in most cases, demonstrating the models’ reliability and accuracy. Table 7 presents evaluation metrics used to assess the performance of the established *ML* and *DL* models. These metrics include the Coefficient of Determination (*R*^2^), Mean Absolute Percentage Error (*MAPE*), Mean Absolute Error (*MAE*), Mean Bias Error (*MBE*), Root Mean Square Error (*RMSE*), A20 Index (A20), Scatter Index (*SI*), and Agreement Index (*d*). Figure 3 through Fig. 5, along with Table 7, compare the predicted and actual values for each model, illustrating their correlation and statistical performance. The performance analysis of the various *ML* and *DL* models provides significant insights into their capabilities, particularly regarding the metrics *R*^2^, *MAPE*, *MAE*, *MBE*, and *RMSE*, A20, *SI*, and *d*.

**Table 7:** Comparison of the developed *ML* and *DL* models.

Algorithm	Train								Test							
	*R* ^2^	*RMSE* (kN/m^2^)	*MAE* (kN/m^2^)	*MBE* (kN/m^2^)	*MAPE* (%)	A20	*SI*	*d*	*R* ^2^	*RMSE* (kN/m^2^)	*MAE* (kN/m^2^)	*MBE* (kN/m^2^)	*MAPE* (%)	A20	*SI*	*d*
*GPR*	1	2.263	0.004	5.835	10.527	100	0.024	1	0.972	11.271	− 2.874	44.206	86.202	88.571	0.195	0.993
*XGBoost*	1	0.655	0	1.525	3.754	100	0.008	1	0.977	13.140	− 3.023	42.622	77.856	80.000	0.176	0.994
*LightGBM*	0.976	7.252	0	33.684	81.323	90.123	0.184	0.994	0.928	26.857	− 19.000	84.034	138.331	51.429	0.314	0.982
*GBM*	1	0.385	0	0.858	3.605	100	0.008	1	0.976	10.649	14.046	43.025	80.407	88.571	0.182	0.993
*RF*	1	0.381	0	0.852	3.605	100	0.008	1	0.959	15.150	10.212	56.659	104.321	80	0.236	0.988
*CATBoost*	1	2.788	0.065	7.460	10.459	100	0.024	1	0.958	15.255	14.323	56.544	105.786	74.286	0.240	0.988
*AdaBoost*	1	3.951	− 1.738	6.574	11.178	97.531	0.025	1	0.942	16.990	27.288	70.612	123.708	68.571	0.280	0.983
*KNN*	1	0.381	0	0.852	3.605	100	0.008	1	0.970	20.226	11.426	56.384	88.452	62.857	0.201	0.992
*BR*	0.997	5.139	0.436	16.936	30.294	95.062	0.069	0.999	0.949	18.555	3.169	64.811	116.181	62.857	0.263	0.985
*DT*	0.999	3.652	0.154	8.416	17.840	96.296	0.040	1	0.958	17.005	6.888	61.894	105.420	71.429	0.239	0.988
*SVM*	0.999	4.609	− 1.730	10.868	15.140	100	0.034	1	0.975	13.131	− 1.024	44.955	81.057	80.000	0.184	0.994
*ANN*	0.999	6.182	− 0.005	11.690	15.893	97.531	0.036	1	0.975	14.375	18.715	45.047	80.854	82.857	0.183	0.993
*DNN*	0.997	9.090	− 3.247	20.319	29.375	90.123	0.066	0.999	0.963	16.729	− 8.023	57.736	98.335	68.571	0.223	0.991
*CNN*	0.993	15.378	13.659	32.338	42.604	72.840	0.096	0.998	0.962	22.058	− 2.048	65.506	99.746	65.714	0.226	0.991
*RNN*	0.966	19.478	2.675	61.367	96.229	70.370	0.218	0.991	0.940	26.210	-12.843	71.617	125.716	62.857	0.285	0.985
*FFNN*	0.994	10.748	6.723	26.519	40.473	91.358	0.092	0.998	0.967	22.331	1.680	59.544	92.994	62.857	0.211	0.992

Gaussian Process Regressor (*GPR*) (Fig. 3(a)) excels with a training *R*^2^ of 0.9996, indicating an excellent fit, and a low *MAPE* of 2.26%, showcasing high accuracy. However, during testing, it shows a decline with a test *R*^2^ of 0.972 and an *MAPE* of 11.27%. The *MAE* increases from 5.84 in training to 44.21 in testing, with an *RMSE* of 86.20, indicating a significant rise in prediction errors. The A20 Index remains strong at 88.57%, indicating that most predictions fall within the acceptable ± 20% error margin. The *SI* of 0.195 suggests moderate scatter, while the Agreement Index (*d*) of 0.993 reflects strong agreement with actual values. Likewise, Extreme Gradient Boost machine (*XGBoost*) (Fig. 3(b)) also performs remarkably, achieving a training *R*^2^ of 0.9999 and an impressively low *MAPE* of 0.65%. Its test performance remains robust with a test *R*^2^ of 0.9771 and an *MAPE* of 13.14%. The *MAE* rises from 1.53 to 42.62, and *RMSE* increases from 3.75 to 77.86, indicating some loss of accuracy. The A20 Index is at 80%, showing a good proportion of predictions within ± 20%. The *SI* of 0.176 suggests low scatter, and the Agreement Index (*d*) of 0.994 indicates strong agreement.

In contrast, Light gradient boosting Machine (*LightGBM*) (Fig. 3(c)), despite a solid training *R*^2^ of 0.9760, struggles in testing with an *R*^2^ of 0.9277 and a high *MAPE* of 26.86%. The *MAE* escalates from 33.68 in training to 84.03 in testing, with an *RMSE* of 138.33. Its A20 Index drops to 51.43%, indicating that a significant number of predictions fall outside the acceptable error margin. *SI* of 0.313 shows increased scatter, and the Agreement Index (*d*) of 0.982 reflects reasonable agreement. Both Gradient Boosting Machine (*GBM*) (Fig. 3(d)) and Random Forest Regressor (*RF*) (Fig. 3(e)) show excellent training performance, with *R*^2^ values of 0.9999 and low *MAPEs* (0.38%). However, they experience increased testing errors, with *GBM* recording a test *R*^2^ of 0.976 and an *MAPE* of 10.65%, while *RF* shows a test *R*^2^ of 0.9589 and an *MAPE* of 15.15%. The *MAE* for *GBM* rises to 43.02, and for *RF*, it increases to 56.66. Their A20 Indices remain high at 88.57% and 80%, respectively, indicating a good proportion of accurate predictions.

Both models have low *SI* values, showing minimal scatter, and strong Agreement Indices (*d*) of 0.993 for *GBM* and 0.988 for *RF*. Categorical Boosting (*CATBoost*) (Fig. 3(f)) delivers a training *R*^2^ of 0.9996 but shows a test *R*^2^ of 0.958 and an *MAPE* of 15.26%. The *MAE* increases from 7.46 to 56.54, with an *RMSE* of 105.79. Its A20 Index is 74.29%, indicating some predictions fall outside the acceptable range, while the *SI* of 0.239 indicates moderate scatter, and the Agreement Index (*d*) of 0.988 suggests strong alignment with actual values. In addition, Ada Boost Regressor (*AdaBoost*) (Fig. 3(g)) achieves a training *R*^2^ of 0.9995 but declines to 0.942 in testing, with an *MAPE* of 16.99%. The *MAE* rises from 6.57 to 70.61, and *RMSE* increases to 123.71. Its A20 Index is at 68.57%, showing a significant number of predictions outside the acceptable margin. The SI of 0.28 indicates moderate scatter, while the Agreement Index (d) of 0.983 reflects good agreement. K-Nearest Neighbors Regression (*KNN*) (Fig. 3(h)) maintains a training *R*^2^ of 0.9999 but drops to 0.9704 in testing, with an *MAPE* of 20.23%. The *MAE* increases from 0.85 to 56.38, and the *RMSE* is at 88.45. The A20 Index is 62.86%, indicating that a considerable proportion of predictions fall outside the ± 20% range. The *SI* is 0.20, showing moderate scatter, and the Agreement Index (*d*) is 0.9919, reflecting strong agreement with actual values.

**Fig. 4:**
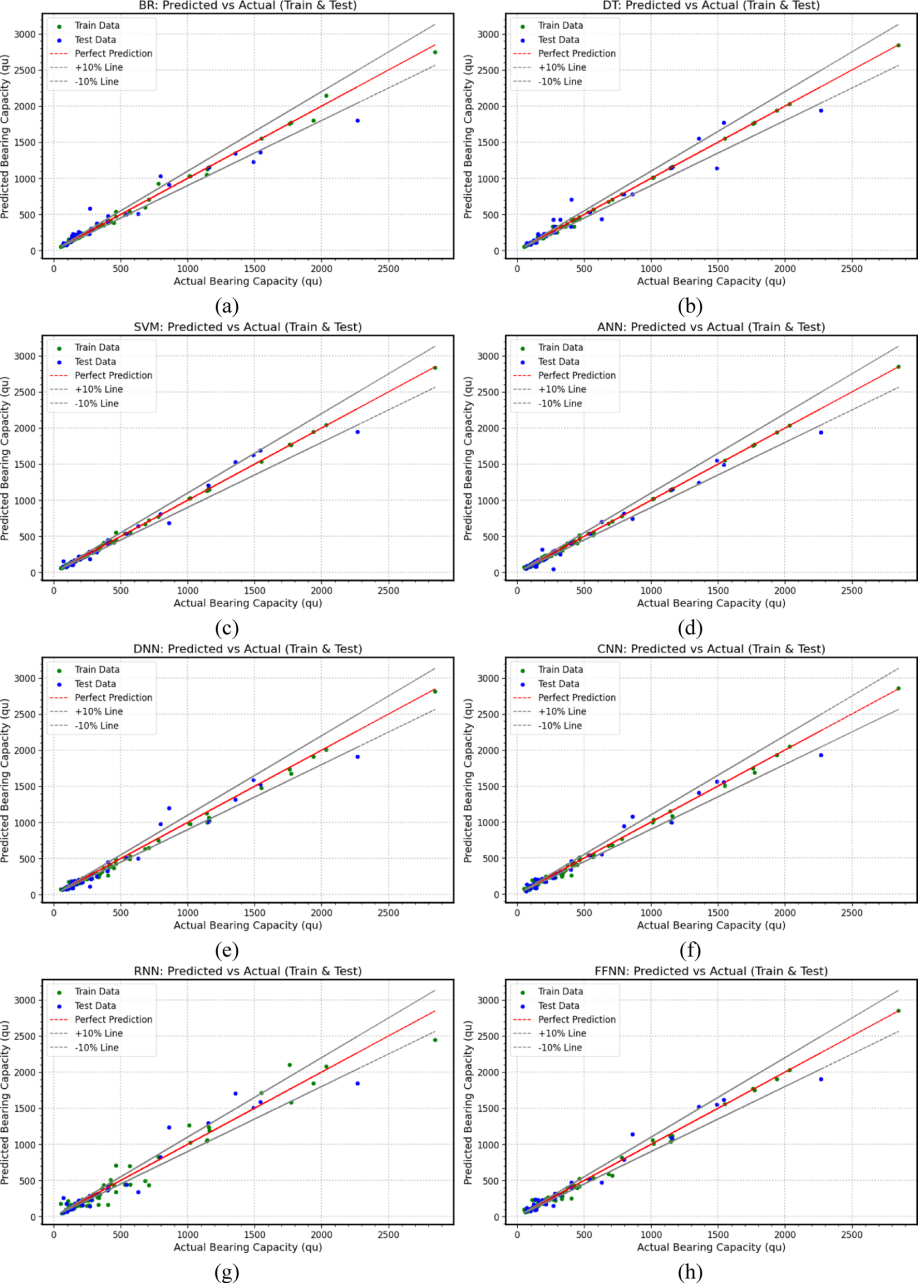
Scatter plots between actual and predicted ultimate bearing capacity values based on (**a**) *BR*, (**b**) *DT*, (**c**) *SVM*, (**d**) *ANN*, (**e**) *DNN*, (**f**) *CNN*, (**g**) *RNN*, (**h**) *FFNN*.

*BR* (Fig. 4(a)) demonstrates strong performance with a training *R*^2^ of 0.9967 and an *MAPE* of 5.14%, indicating a good fit to the training data. In the test set, it also performs well, achieving a test *R*^2^ of 0.9490 and an *MAPE* of 18.56%. However, the *MAE* increases from 16.94 to 64.81, and the *RMSE* rises from 30.29 to 116.18, suggesting a significant increase in prediction errors. Its A20 Index is 62.86%, the *SI* is 0.263, and the *d* is 0.9851, showing a significant number of predictions outside the acceptable margin. On the other hand, Decision Trees (*DT*) (Fig. 4(b)) achieve excellent training metrics with an *R*^2^ of 0.9988 and a low *MAPE* of 3.65%. It also maintains a strong test performance, with a test *R*^2^ of 0.9580 and an *MAPE* of 17.00%. The *MAE* increases from 8.42 to 61.89, and the *RMSE* rises from 17.84 to 105.42. With an A20 Index of 71.43%, a low *SI* of 0.239, and a high Agreement Index (*d*) of 0.9884, Decision Trees indicate some predictions fall outside the acceptable range. In addition, Support Vector Machines (*SVM*) (Fig. 4(c)) perform exceptionally well, with a training *R*^2^ of 0.9992 and an *MAPE* of 4.61%. Its test performance is among the best, achieving a high test *R*^2^ of 0.9752, an *MAPE* of 13.13%, an *MAE* of 44.96, and an *RMSE* of 81.06. The A20 Index stands at 80%, the *SI* is 0.184, and the Agreement Index (*d*) is 0.9937, highlighting its strong predictive capabilities and low scatter. Neural network models also showcase strong performance.

Artificial Neural Networks (*ANN*) (Fig. 4(d)) have a training *R*^2^ of 0.9991 and an *MAPE* of 6.18%. They generalize well to the test set, achieving a test *R*^2^ of 0.9753 and an *MAPE* of 14.37%. The *MAE* is 45.05, the *RMSE* is 80.85, the A20 Index is 82.86%, the *SI* is 0.183, and the Agreement Index (*d*) is 0.9934, indicating a highly reliable model. The Deep Neural Network (*DNN*) (Fig. 4(e)) model also performs well, with training metrics of *R*^2^ = 0.9969 and MAPE = 9.09%. It maintains a respectable test *R*^2^ of 0.9635, though with a higher *MAPE* of 16.73%. Its A20 Index of 68.57% and *SI* of 0.223 indicate a significant number of predictions outside the acceptable margin.

Both Convolutional Neural Networks (*CNN*) (Fig. 4(f)) and Feedforward Neural Networks (*FFNN*) (Fig. 4(g)) exhibit strong performance. CNN, despite a lower training *R*^2^ of 0.9934 and a higher MAPE of 15.38%, achieves a good test *R*^2^ of 0.9624 and an *MAPE* of 22.06%. *FFNN* shows similar results, with a training *R*^2^ of 0.9941 and an *MAPE* of 10.75%, as well as a test *R*^2^ of 0.9673 and an *MAPE* of 22.33%. Lastly, the Recurrent Neural Network (*RNN*) (Fig. 4(h)) has a significantly lower training *R*^2^ of 0.9664 and a high *MAPE* of 19.48%. Its performance declines in testing as well, recording a test *R*^2^ of 0.9403 and a high *MAPE* of 26.21%, indicating it is less effective at this prediction task compared to the other models.

**Fig. 5:**
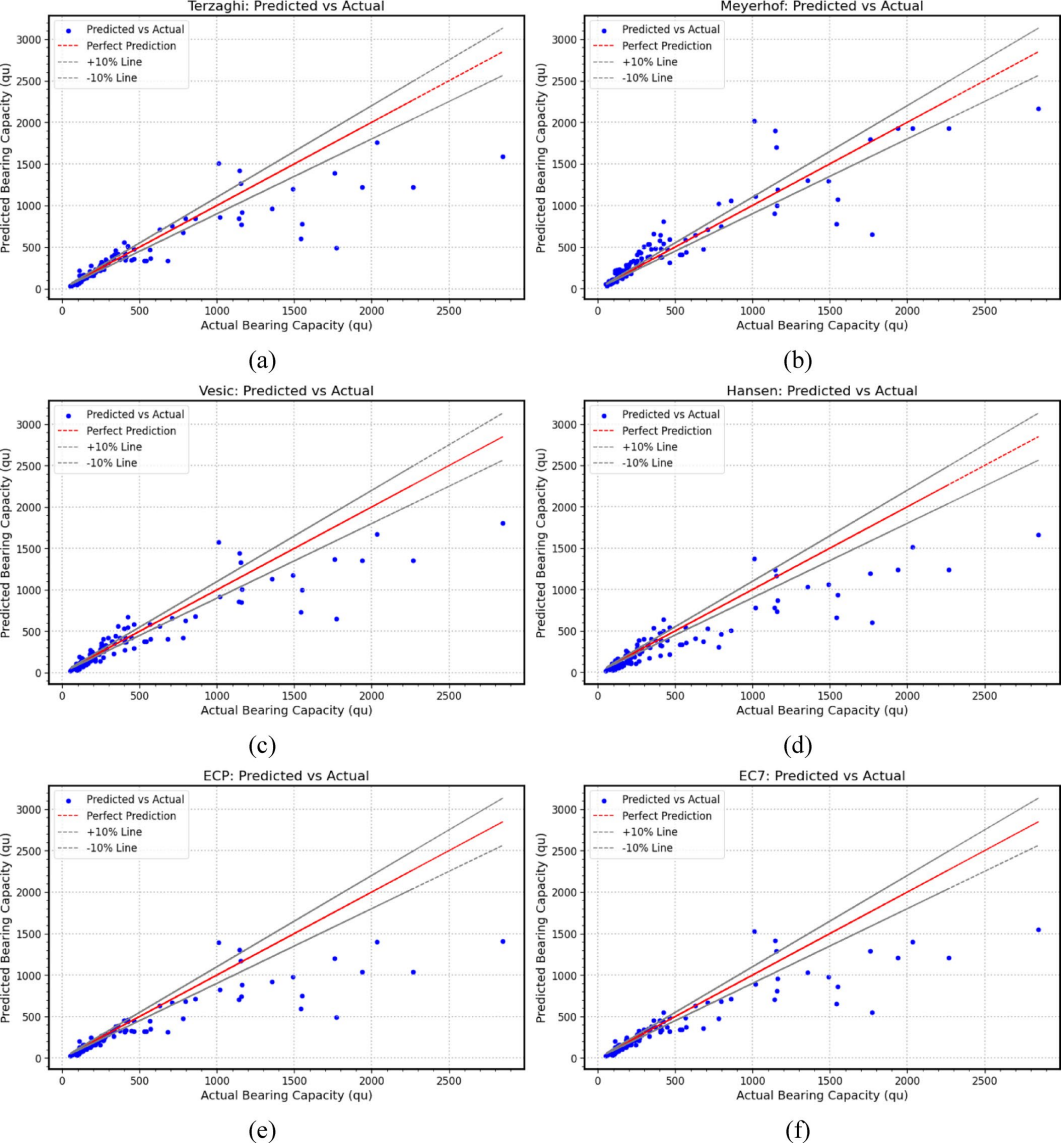
Scatter plots between actual and predicted ultimate bearing capacity values based on (**a**) Terzaghi, (**b**) Meyerhof, (**c**) Vesić, (**d**) Hansen, (**e**) *EC*7, (**f**) *ECP*.

The ultimate bearing capacity predictions of footings by the proposed equations were compared with existing code formulas, including *Terzaghi*, *Meyerhof*, *Vesić*, *Hansen*, *ECP*, and *EC*7, for different types of footings in sand. *Terzaghi* (Fig. 5(a)), *Meyerhof* (Fig. 5(b)), *Vesić* (Fig. 5(c)), *Hansen* (Fig. 5(d)), *ECP* (Fig. 5(e)), and *EC*7 (Fig. 5(f)) exhibited comparatively the lowest values, with *R*^2^ values ranging from 0.684 to 0.82 and CC values from 0.9 to 0.921, respectively. These models also exhibited the highest errors (*RMSE*: 221.65 to 293.49 kN/m^2^, *MAE*: 115.88 to 142.84 kN/m^2^, *MBE*: − 23.08 to 116.30 kN/m^2^) with an *MAPE* of more than 19.63%.

The analysis reveals that advanced models generally outperform traditional code formulas. Models such as *GPR*, *XGBoost*, *GBM*, *DT*, *SVM*, and *ANN* stand out as top performers, demonstrating excellent training and testing metrics. For instance, *XGBoost* achieves an exceptionally high training *R*^2^ of 0.9999 and a low *MAPE* of 0.65%, which translates to a robust test performance with an *R*^2^ of 0.9771 and an *MAE* of 42.62. Similarly, *GPR* and *SVM* also maintain high test *R*^2^ values of 0.972 and 0.9752, respectively, along with low error metrics and high A20 Index values, indicating reliability and strong generalization. In contrast, models like *LightGBM*, *RNN*, *AdaBoost*, and *BR* show a more significant performance drop from training to testing, with *LightGBM* recording a test *R*^2^ of 0.9277 and a high *MAPE* of 26.86%, while *RNN’s* performance declines to a test *R*^2^ of 0.9403 and a *MAPE* of 26.21%, indicating that a significant number of predictions fall outside the acceptable error margin. The traditional formulas, including those from *Terzaghi*, *Meyerhof*, *Vesić*, *Hansen*, *ECP*, and *EC*7, exhibit the weakest performance overall, with the lowest *R*^2^ values (ranging from 0.684 to 0.82) and the highest error metrics (*RMSE* from 221.65 to 293.49 and *MAE* from 115.88 to 142.84), confirming that *ML* and *DL* approaches are significantly more accurate for this task.

While the *GPR*, *XGBoost*, *GBM*, *DT*, and *SVM* models demonstrate notably better performance than other models, deriving a clear design formula from them is difficult. Although *DL* models can yield precise and explicit formulas for strength prediction, applying these networks in engineering design may be impractical because of the complex and lengthy formulas they produce^100^.

### The regression error characteristics curve

**Fig. 6:**
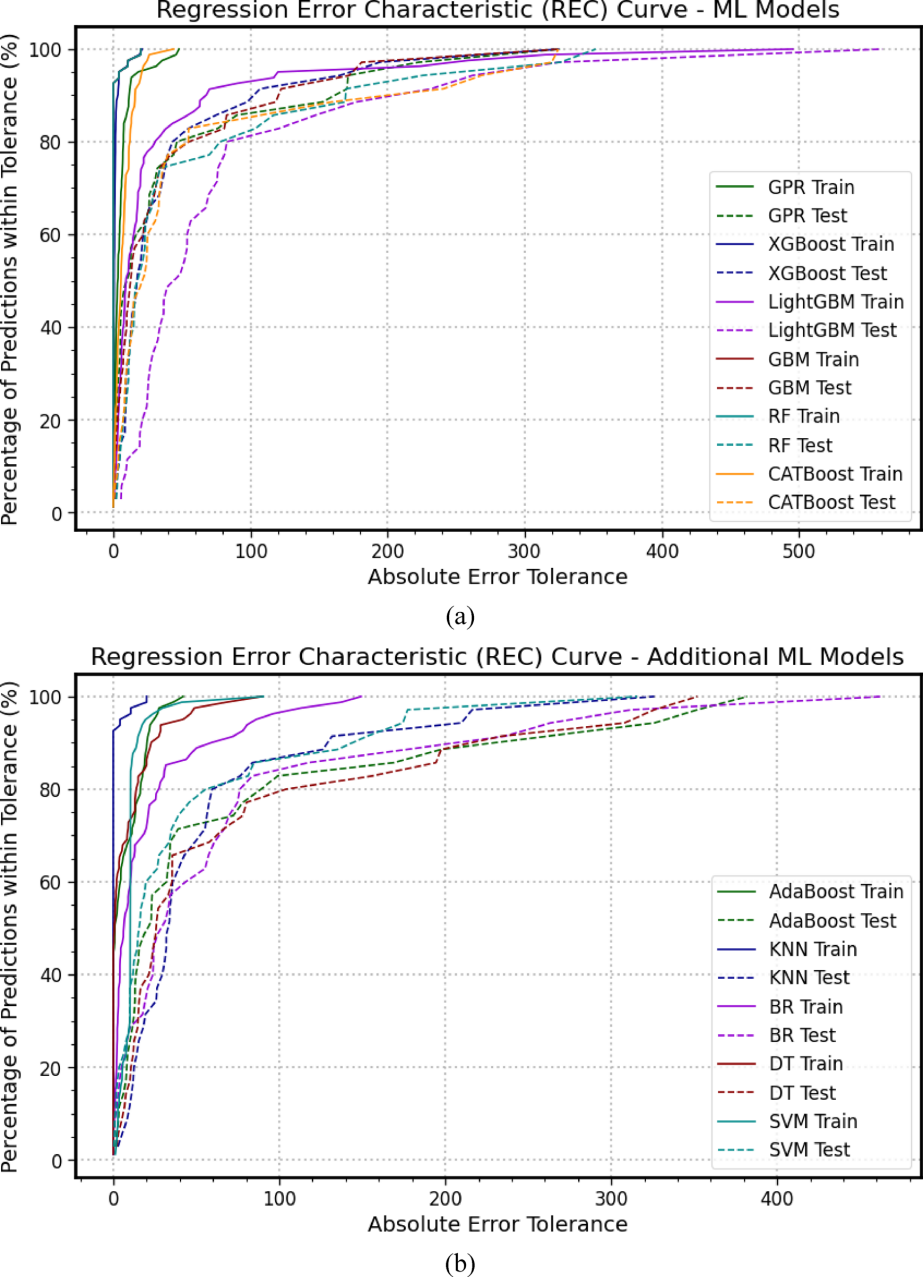

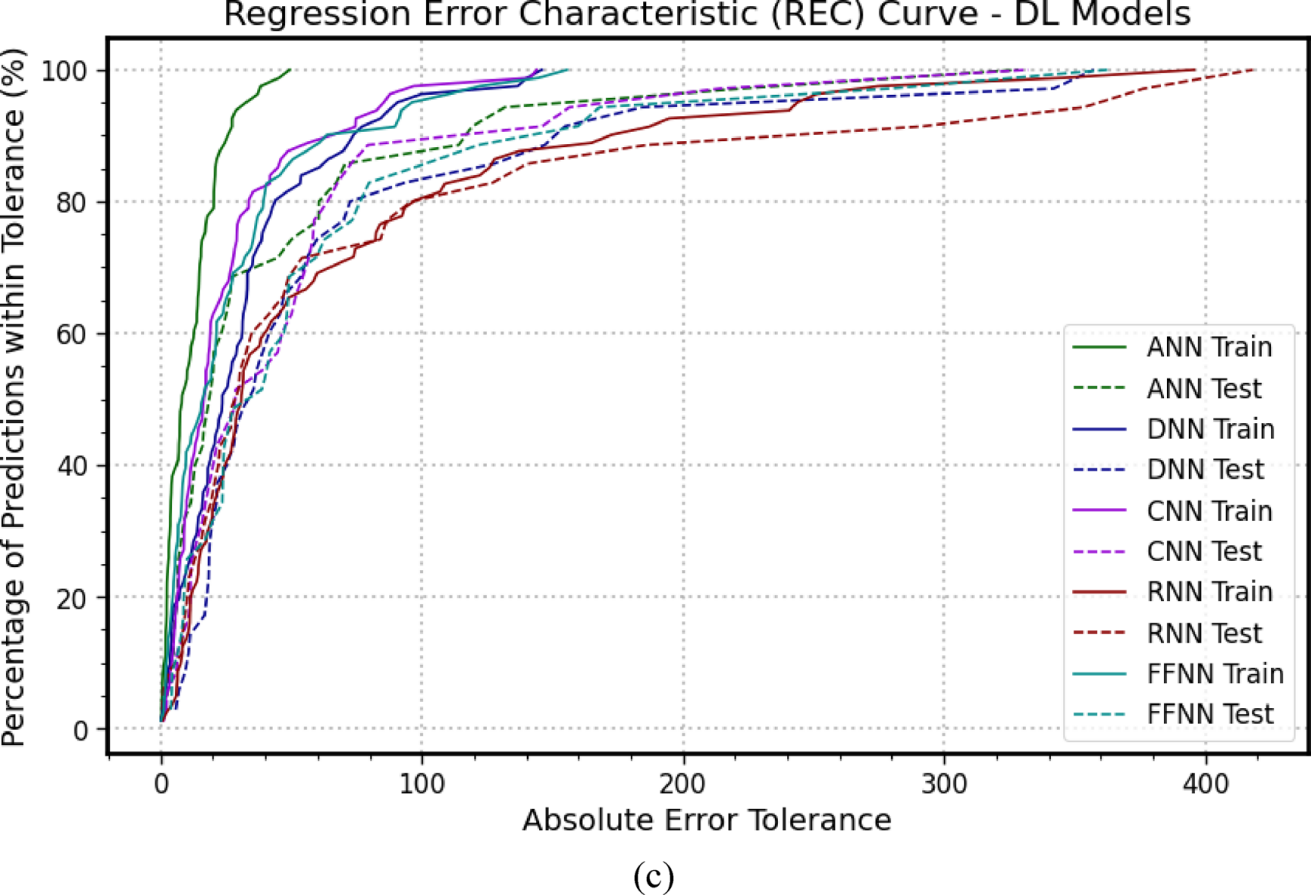
REC curves illustrate the performance of the adopted models during the training and testing (**a**) *GPR*, *XGBoost*, *LightGBM*, *GBM*, *RF*, *CATBoost*; (**b**) *AdaBoost*, *KNN*, *BR*, *DT*, *SVM*; (**c**) *ANN*, *DNN*, *CNN*, *RNN*, *FFNN*.

Figure 6 presents the Regression Error Characteristics (*REC*) curve, which is used to evaluate the predictive robustness of the models during both the training and testing phases^73,78^. The curves illustrate the accuracy of each model in estimating the ultimate bearing capacity. In the training phase, shown in Fig. 6(a), *XGBoost*, *GBM*, and *RF* exhibited the highest predictive accuracy, with *CATBoost* and *GPR* following closely behind. In contrast, *LightGBM* demonstrated the weakest performance. During the testing phase, *XGBoost* and *GBM* maintained their position as the most accurate models. *GPR*, *RF*, and *CATBoost* performed similarly, while *LightGBM* again showed the least robust performance, indicating a consistent lack of reliability. As shown in Fig. 6(b) for the training phase, the *KNN* model achieved the highest predictive accuracy, followed closely by *AdaBoost*, *SVM*, and *DT*. The *BR* model, however, lagged with the poorest performance. In the testing phase, all models performed comparably, except for *BR*, which consistently displayed the lowest performance, indicating a persistent lack of robustness. In the training phase illustrated in Fig. 6(c), the ANN model produced the highest predictive accuracy, followed by *DNN*, then *CNN* and *FFNN*, while the *RNN* model exhibited the weakest performance. During the testing phase, all models performed similarly, except for *RNN*, which again showed the lowest performance, suggesting a continual lack of robustness in the *RNN* model. These results reinforce the findings previously presented.

### Score analysis

**Fig. 7:**
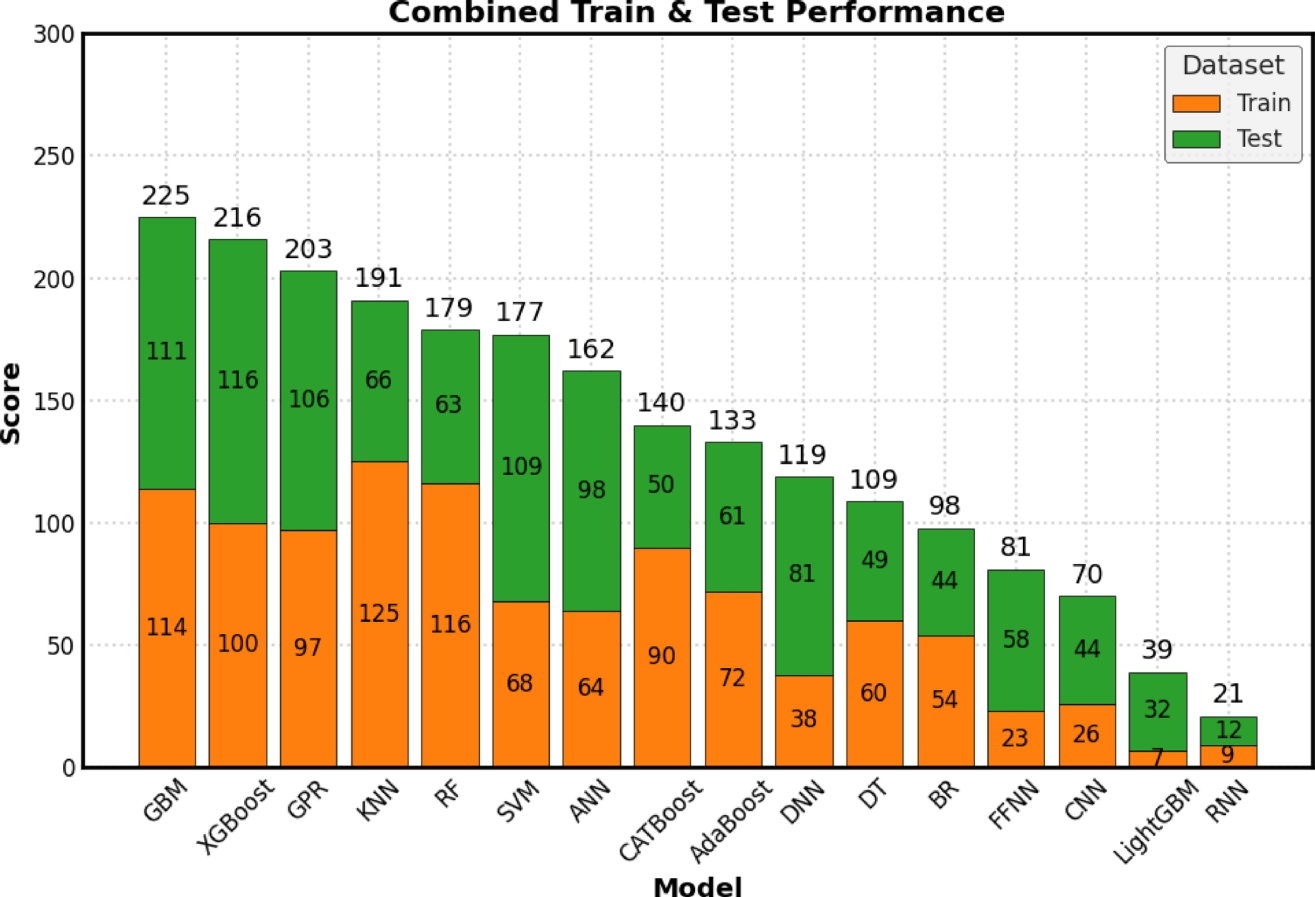
Score analysis indicates the performance of 16 *ML* and *DL* models.

The score analysis presents a detailed ranking of 16 machine learning algorithms based on their performance in both training and testing datasets, as illustrated in Fig. 7. For each algorithm, a composite score was calculated by summing its ranks across 16 key performance metrics (eight from the training set and eight from the testing set). These metrics include the Coefficient of Determination (*R*^2^), Mean Absolute Percentage Error (*MAPE*), Mean Absolute Error (*MAE*), Mean Bias Error (*MBE*), Root Mean Square Error (*RMSE*), A20 Index (A20), Scatter Index (*SI*), and Agreement Index (*d*). A higher total score indicates better overall performance. This analysis includes various performance metrics derived from the following 16 machine learning algorithms: *GPR*, *XGBoost*, *LightGBM*, *GBM*, *RF*, *CATBoost*, *AdaBoost*, *KNN*, *BR*, *DT*, *SVM*, *ANN*, *DNN*, *CNN*, *RNN*, and *FFNN*. The final score for each algorithm reflects the sum of its ranks across all 16 metrics.

The Gradient Boosting Machine (*GBM*) emerged as the top performer, excelling in both the training and testing datasets with a score of 225. *XGBoost* and Gradient Boosting Regression (*GPR*) closely followed in second and third places, with scores of 216 and 203, respectively. These models demonstrated strong robustness and excellent generalization capabilities for new, unseen data. In contrast, the Recurrent Neural Network (*RNN*), with a score of 21, and *LightGBM*, with a score of 39, experienced significant drops in performance from the training to the testing phase, indicating potential overfitting issues.

The ranking methodology adhered to several key principles. Each of the eight performance metrics was evaluated based on its ideal value (higher *R*^2^), while lower *RMSE* values are desirable. For the Mean Bias Error (*MBE*), which can take both positive and negative values, models were ranked according to the absolute values of their scores to ensure fairness between over-prediction and under-prediction biases. Additionally, a two-step sorting process was employed to break ties: first by metric value and then alphabetically by the algorithm’s name. An algorithm’s performance on the training dataset reflects its ability to learn effectively from the data provided. The ranks for each algorithm across the eight metrics for both training and testing sets ranged from 16 (best) to 1 (worst).

This analysis provided valuable insights into model behavior. A clear distinction emerged between models that merely memorized the training data and those that generalized effectively. Models that exhibited significant declines in performance from training to testing displayed signs of overfitting. The top three algorithms, *GBM* (225), *XGBoost* (216), and GPR (203), consistently performed well across both datasets, making them the most reliable choices for predictive tasks. The results are consistent with the previous findings.

### Performance assessment via Taylor diagram

**Fig. 8:**
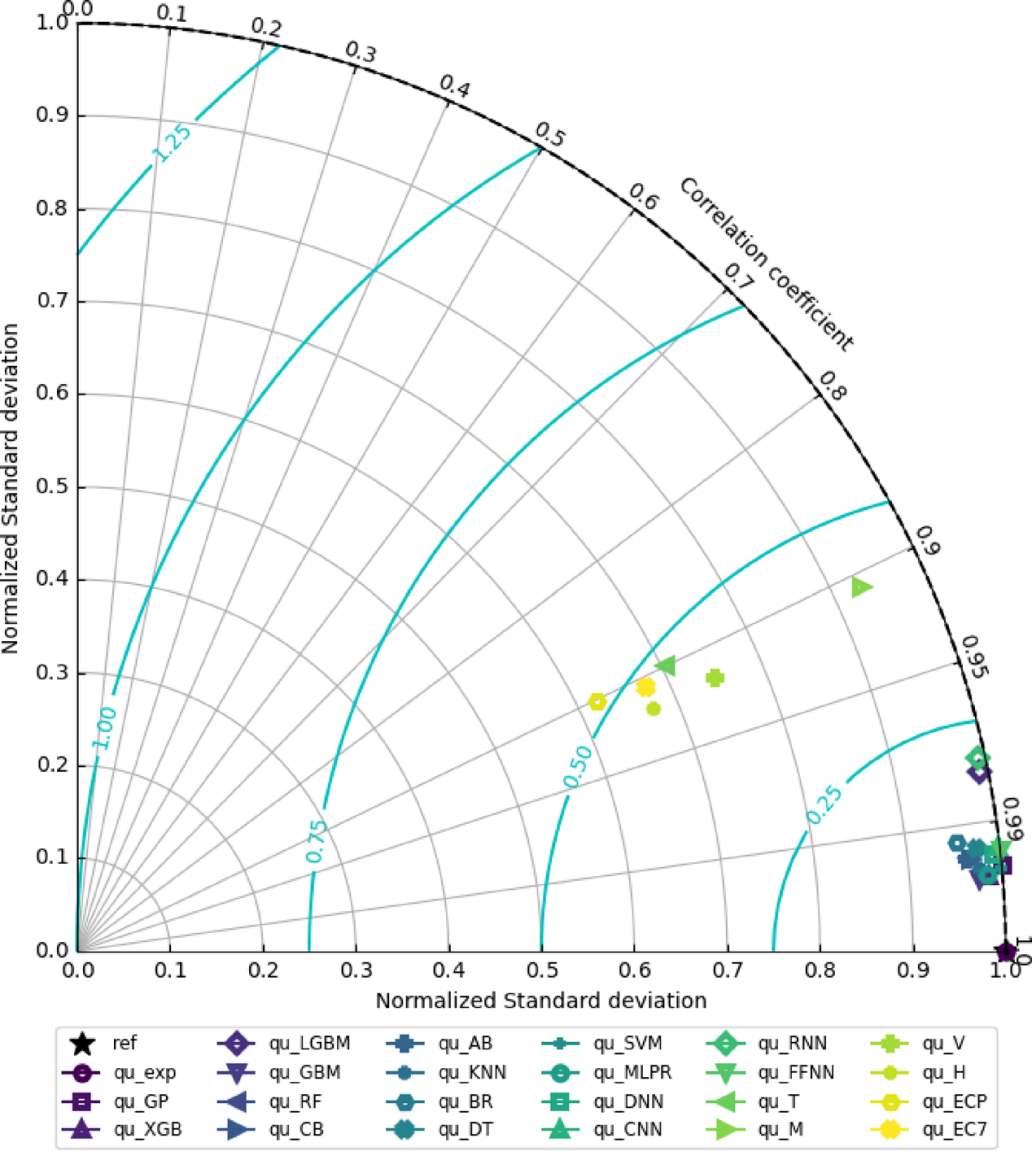
Taylor diagram indicating the performance of 23 models.

Using a Taylor diagram (Fig. 8), the study compared machine learning, deep learning models, and traditional equations to experimental footing bearing capacity, evaluating normalized standard deviation, Correlation Coefficient (*CC*), and Normalized Root Mean Square Error (*NRMSE*). The machine and deep learning models consistently outperformed the traditional equations. Specifically, they demonstrated high *CC* values (mostly > 0.99), low *NRMSE* (mostly < 0.13), and normalized standard deviations exceeding 0.95, while the traditional equations showed lower *CC* (~ 0.9), higher *NRMSE* (~ 0.5), and lower normalized standard deviations (~ 0.65). *LightGBM* and *RNN* exhibited slightly lower performance within the machine learning models. As observed, *XGBoost* and *GBM* models demonstrated slightly better overall prediction accuracy than the other models.

### Feature importance analysis

**Fig. 9:**
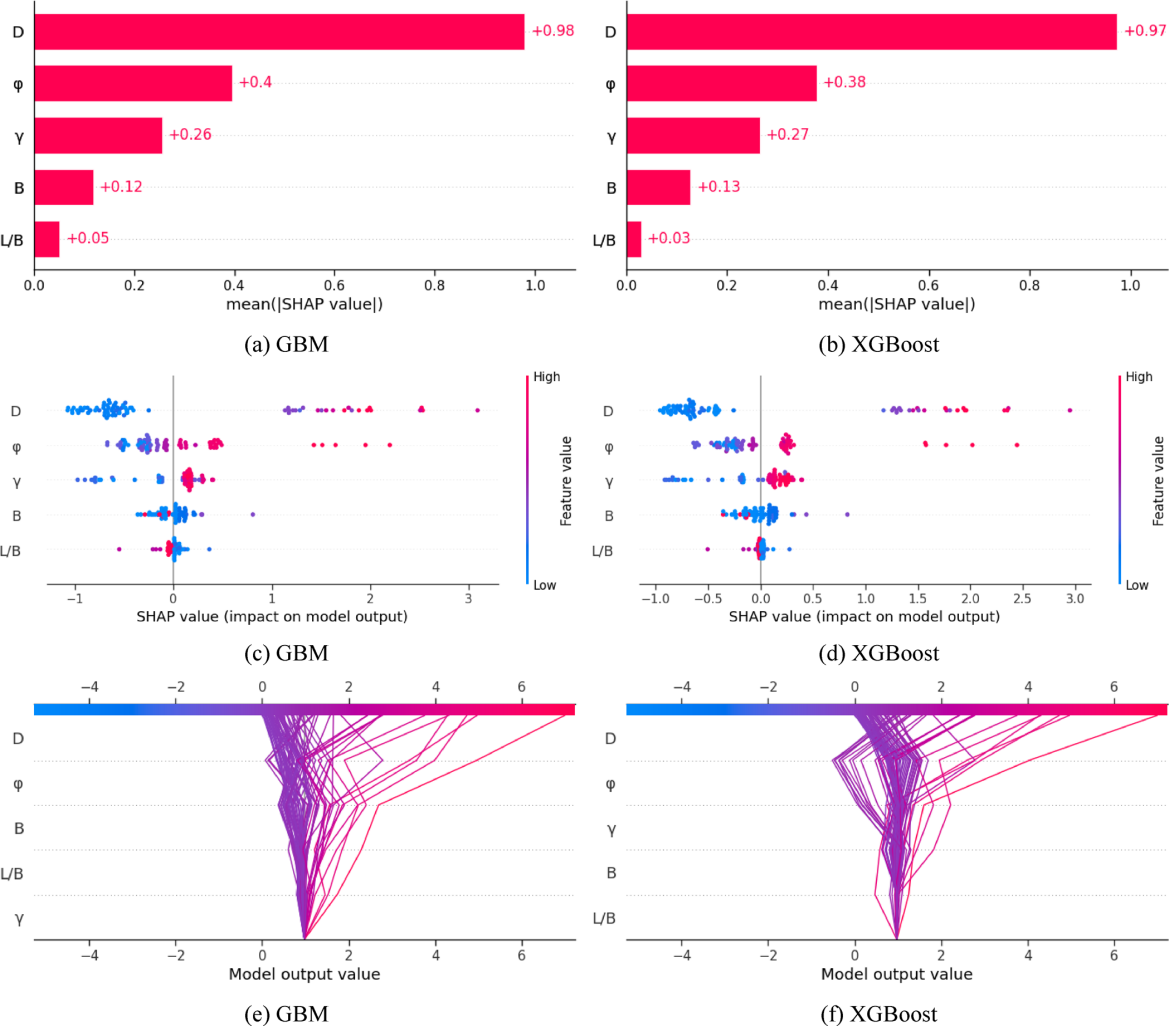
SHAP decision plots for footings database.

To explore how input parameters influence the ultimate bearing capacity of footings with different shapes on cohesionless soil, this study utilized Shapley Additive Explanation (*SHAP*) analysis. Figure 9(a, f) present the *SHAP* analysis for *GBM* and *XGBoost* models. Specifically, Fig. 9(a, b) summarize the effects and relative importance of each feature on the model’s bearing capacity predictions. Figure 9(c, d) present the *SHAP* feature importance for each input variable, where positive values indicate a positive correlation with bearing capacity and negative values a negative correlation. Figure 9(e, f) feature *SHAP* decision plots that illustrate the intricate decision-making processes of these machine learning models, offering insights into the global prediction behavior depicted in the summary plots. Analysis of the *SHAP* features reveals a high degree of similarity between the *GBM* and *XGBoost* models. Foundation depth is the most important design parameter, with the angle of internal friction having a significant impact, followed by soil unit weight and foundation width. In contrast, the length-to-width ratio has the least impact.

The relationships identified by the machine learning models are consistent with established engineering principles, reinforcing their value as tools for improving design intuition and practical decision-making. For example, the positive correlation between foundation depth and ultimate bearing capacity aligns with geotechnical theory, as greater foundation depth inherently supports a higher ultimate bearing capacity before failure. Similarly, the effects of the angle of internal friction and soil unit weight on ultimate bearing capacity predictions are well recognized within geotechnical engineering. The significant role of foundation dimensions (width and length-to-width ratio) in these predictions underscores their importance in enhancing load resistance and reducing footing settlement, a concept well understood in geotechnical engineering. The insights generated by the model regarding these relationships improve interpretability and provide geotechnical engineers with a deeper understanding of how individual parameters affect geotechnical behaviour, ensuring that the model functions as a valuable tool that connects advanced computation with fundamental engineering principles.

## Conclusions

In this study, we evaluated the performance of various machine learning (*ML*) and deep learning (*DL*) models for predicting the ultimate bearing capacity (*q*_*u*_) of shallow foundations in cohesionless soil using a dataset of 116 footing experiments. The dataset comprised 64 small-scale and 52 large-scale experiments. The following are the key findings and contributions of this research.

Results demonstrate that advanced *ML* and *DL* models are significantly superior to traditional theoretical equations (*Terzaghi*, *Meyerhof*, *Vesić*, *Hansen*, *ECP*, *EC*7), consistently providing predictions that align closely with experimental data. Ensemble methods like *XGBoost* and *GBM* emerged as the top performers, with *XGBoost* showing the highest test *R*^2^ (0.9771) and the lowest prediction errors, while *GBM* achieved the lowest test *MAPE* (10.65%) and the highest A20 Index (88.57%). The *SHAP* analysis reinforced these findings by revealing that foundation depth (*D*) was the most influential parameter, which aligns with established geotechnical principles. This consistency enhances the models’ interpretability and trustworthiness for engineering applications.

This research is primarily based on a dataset that, while including large-scale experiments, is still limited in its overall size and diversity. The performance of the developed models is highly dependent on the quality and representativeness of the specific data used. Consequently, their effectiveness may vary when applied to different data or conditions outside the scope of this study, and the models’ generalizability needs to be further assessed.

The primary advantage of this study is the significant improvement in the accuracy and reliability of predicting shallow foundation bearing capacity compared to conventional methods. The use of explainable AI techniques like *SHAP* analysis provides valuable insights into the relationships between input parameters and the models’ predictions, bridging the gap between advanced computational methods and fundamental engineering understanding. Furthermore, making the developed Python tool available upon request will facilitate the reproduction of our methodology and support future research in the field.

To validate and broaden the applicability of these findings, future research should focus on a few key areas. First, it is essential to validate these models using full-scale experimental data. Second, expanding the dataset with more extensive and diverse experimental data will enhance the reliability and generalizability of the models. Finally, exploring more advanced feature engineering techniques and continuing to improve the interpretability of complex models are promising avenues for future work.^101^–^103^

## Data Availability

All data supporting the findings of this study are available from the corresponding author upon reasonable request.

## Abbreviations

*AI*: Artificial intelligence
*ML*: Machine learning
*DL*: Deep learning
*SHAP*: SHapley additive exPlanations
*GPR*: Gaussian process regressor
*XGBoost*: Extreme gradient boost machine
*LGBM*: Light gradient boosting machine
*GBM*: Gradient boosting machine
*RF*: Random forest regressor
*CatBoost*: Categorical boosting
*AdaBoost*: Ada boost regressor
*KNN*: K-Nearest neighbors regression
*BR*: Bagging regressor (Bootstrap aggregating)
*DT*: Decision tree regressor
*SVR*: Support vector machine
*ANN*: Artificial neural network (*MLP*)
*DNN*: Deep neural network
*CNN*: Convolutional neural network
*FFNN*: Feed forward neural network
*RNN*: Recurrent neural networks
*R*^2^: Coefficient of determination
*MAPE*: Mean absolute percentage error
*MBE*: Mean bias error
*MAE*: Mean absolute error
*RMSE*: Root mean square error
*NRMSE*: Normalized root mean square error
*CC*: Correlation coefficient
A20: A20 Index
*SI*: Scatter Index
*d*: Agreement Index
*q*_*u*_: Ultimate bearing capacity
*c*: Soil cohesion
$\gamma$: Effective unit weight of the soil
*B*: Footing width
*L*: Footing length
*D*: Foundation depth
${N}_{c}$, ${N}_{q}$, ${N}_{\gamma }$: Non-dimensional bearing capacity factors
$\varphi$: Internal friction angle
${s}_{c}$, ${s}_{q}$, ${s}_{\gamma }$: Non-dimensional shape factors
${d}_{c}$, ${d}_{q}$, ${d}_{\gamma }$: Non-dimensional depth factors
${K}_{p}$: Passive earth pressure
